# Tuning the Ferroelectric Response of Sandwich-Structured Nanocomposites with the Coordination of Ba_0.6_Sr_0.4_TiO_3_ Nanoparticles and Boron Nitride Nanosheets to Achieve Excellent Discharge Energy Density and Efficiency

**DOI:** 10.3390/polym15173642

**Published:** 2023-09-04

**Authors:** Zhihui Yi, Zhuo Wang, Dan Wu, Ying Xue

**Affiliations:** Shaanxi Key Laboratory of Green Preparation and Functionalization for Inorganic Materials, School of Materials Science and Engineering, Shaanxi University of Science & Technology, Xi’an 710021, China

**Keywords:** PVDF, nanocomposites, energy storage, Ba_0.6_Sr_0.4_TiO_3_ NPs, dielectrics

## Abstract

With the rapid development of new electronic products and sustainable energy systems, there is an increasing demand for electrical energy storage devices such as electrostatic capacitors. In order to comprehensively improve the dielectric, insulating, and energy storage properties of PVDF-based composites, sandwich-structured composites were prepared by layer-by-layer solution casting. The outer layers of the sandwich structure composite are both PVDF/boron nitride nanosheet composites, and the middle layer is a PVDF/Ba_0.6_Sr_0.4_TiO_3_ nanoparticles composite. The structural and electrical properties of the sandwich-structured composites were characterized and analyzed. The results show that when the volume percentage of Ba_0.6_Sr_0.4_TiO_3_ nanoparticles in the middle layer of the sandwich structure composite is 1 vol.%, the dielectric properties are significantly improved. Its dielectric constant is 8.99 at 10 kHz, the dielectric loss factor is 0.025, and it has better insulating properties and resistance to electrical breakdown. Benefiting from the high breakdown electric field strength and the large maximum electrical displacement, the sandwich-structured composites with 1 vol.% and Ba_0.6_Sr_0.4_TiO_3_ nanoparticles in the middle layer show a superior discharge energy density of 8.9 J/cm^3^, and excellent charge and discharge energy efficiency of 76%. The sandwich structure composite achieves the goal of simultaneous improvement in breakdown electric field strength and dielectric constant.

## 1. Introduction

Driven by the rapidly depleted background of limited fossil fuels, a great deal of research has been focused on the exploration of renewable, green, and sustainable energy sources and advanced energy storage technologies [1,2]. Compared with batteries, fuel cells, and supercapacitors, dielectric capacitors stand out among various energy storage devices because of their ultra-fast charge-discharge speed, high power density, and longer cycle times [3]. These key properties have greatly facilitated the widespread application of dielectric capacitors in modern electronic and electrical industries, including but not limited to portable electronic devices, computing systems, high-power pulsed lasers, and smart grids.

The charge and discharge energy density of a dielectric capacitor are determined by the dielectric material. In order to improve the energy storage density of capacitors, the method of compounding organic/inorganic materials can be used to prepare polymer-based composite dielectric materials [4]. As a new type of dielectric material, polymer-based composite dielectric materials can combine the unique thermal, mechanical, and electrical properties of inorganic materials with the good processability and high breakdown strength of polymer materials [5]. According to the calculation Formula (1) of the energy storage density of dielectric material [6]:

(1) $$U=\int_{0}^{D_{max}}EdD=\int_{0}^{P_{max}}EdP$$

The energy storage density value of dielectric material is determined by the electric field strength and electric displacement in the charge-discharge curve [7]. Therefore, in order to obtain materials with high energy storage density, on the one hand, it is necessary to increase the breakdown electric field strength of the dielectric material as much as possible [8]. In order to improve the dielectric constant of dielectric materials, high dielectric ceramic fillers are often introduced into polymers in large quantities [9,10,11]. Unfortunately, due to the huge difference between the dielectric constant of the high dielectric filler and the polymer matrix, the local electric field distribution of the composite material is uneven, and the breakdown strength of the composite material is greatly reduced [12,13].

In order to improve the high electric field resistance of composite materials, a large number of inorganic two-dimensional materials such as montmorillonite nanosheets, zirconia nanosheets, sodium bismuth titanate nanosheets, potassium sodium niobate nanosheets, and kaolin nanosheets are used as inorganic fillers in the polymer matrix [14,15,16]. The insulating two-dimensional inorganic material forms a curved channel when the electrical dendrites inside the composite material evolve in the polymer matrix, which increases the tortuosity of the breakdown path, thereby improving the breakdown field strength of the composite material [17,18]. The two-dimensional sheet-like inorganic materials can generate more traps, which can effectively scatter the charges injected into the composite material [19]. The two-dimensional sheet-like inorganic material reduces the leakage current value of the material, thereby reducing the conductivity loss, which is beneficial to the improvement in the energy storage density and the charge-discharge efficiency of the dielectric material [20]. Although the addition of these 2D inorganic materials improves the breakdown field strength of composite dielectrics, the dielectric constants of the resulting composites are usually not high, and some are even lower than pure ferroelectric fluoropolymers [21]. The low dielectric constant limits the improvement in the energy storage density of composite materials to a greater extent [22].

To solve the above problems, composite materials with a sandwich structure have been designed [23,24,25]. Compared with single-layer composite films, composite films with a sandwich structure are expected to solve the contradiction that composite materials composed of inorganic and organic materials cannot simultaneously achieve improved breakdown strength and improved high dielectric constant [26,27]. Layers of materials with high dielectric constants and high breakdown strength are stacked layer by layer to form sandwich-structured composites [28,29]. In sandwich-structured composites, different functional layers exhibit synergistic advantages in enhancing energy density [30,31].

In this paper, polyvinylidene fluoride (PVDF) was chosen as the polymer matrix of the sandwich-structured composites. At a frequency of 100 Hz, the dielectric constant of PVDF is close to 10, which is beneficial to the improvement in the dielectric constant and dielectric polarization strength of the composite material [32]. Ba_0.6_Sr_0.4_TiO_3_ nanoparticles (Ba_0.6_Sr_0.4_TiO_3_ NPs, BST NPs) with a high dielectric constant [33], and two-dimensional boron nitride nanosheets (BNNS) are used as inorganic fillers in the middle and outer layers, respectively. Compared with other inorganic particles, barium strontium titanate nanoparticles have a higher dielectric constant, which contributes more to the improvement in the dielectric constants of composite materials. BNNS has high forbidden bandwidth (6 eV), high insulation, high breakdown electric field strength (800 kV/mm), and high thermal conductivity [34,35,36]. The addition of BNNS is expected to improve the insulating properties of the materials and thus improve the dielectric energy storage properties of the composites.

## 2. Experimental Section

### 2.1. Raw Materials

Boron nitride nanosheets (BNNS), acetylacetone, glacial acetic acid, N, N-dimethylformamide (DMF), anhydrous ethanol, boron nitride nanosheets, dopamine hydrochloride (DA-HCl), oxalic acid dihydrate (HC_2_O_4_·2H_2_O), barium nitrate (Ba(NO_3_)_2_), strontium nitrate (Sr(NO_3_)_2_), and tetrabutyl titanate were purchased from Sinopharm Chemical Reagent Co., Ltd., Shanghai, China. Polyvinylidene fluoride (PVDF) was obtained from Sigma-Aldrich Co., LLC, St. Louis, MO, USA.

### 2.2. Fabrication of Ba_0.6_Sr_0.4_TiO_3_ Nanoparticles

To prepare Ba_0.6_Sr_0.4_TiO_3_ nanoparticles, Ba(NO_3_)_2_, tetrabutyl titanate, and Sr(NO_3_)_2_ were used as the main raw materials, oxalic acid dihydrate was used as precipitant, and a mixed solution (deionized water and ethanol) was used as solvent. The specific experimental steps were as follows: A beaker was poured with 0.1 mol of HC_2_O_4_·2H_2_O. Amounts of 150 mL of distilled water and 50 mL of absolute ethanol were measured as solvents. Under the condition of heating in a water bath, the liquid in the beaker was stirred, so that HC_2_O_4_·2H_2_O was completely dissolved, and the mixed solution was marked as Mixture A. An amount of 0.05 mol of tetrabutyl titanate was dissolved in 100 mL of absolute ethanol, heated and stirred to mix well, and this mixed solution was marked as Mixture B. Amounts of 0.02 mol Sr(NO_3_)_2_ and 0.03 mol Ba(NO_3_)_2_ were dissolved in 100 mL of distilled water, heated and stirred until Sr(NO_3_)_2_ and 0.03 mol Ba(NO_3_)_2_ were completely dissolved, and the mixed solution was marked as a Mixture C. Mixture B and Mixture A were mixed, and after being stirred evenly, ammonia water was added to adjust the pH to about 3.5 to obtain Mixture D. Under the condition of heating to 80 °C in a water bath, Mixture C was slowly added dropwise to Mixture D, and then stirred for 2 h to separate the compounds. After the complete reaction, the resulting mixed solution was aged for 24 h. After being aged for 24 h, the precursor of Ba_0.6_Sr_0.4_TiO_3_ powder was centrifuged and washed. Then, the precursor of Ba_0.6_Sr_0.4_TiO_3_ powder was dried in a drying oven at 100 °C for 6 h and calcined in a muffle furnace at 800 °C. After the Ba_0.6_Sr_0.4_TiO_3_ powder was ground by a ball mill for 12 h, the Ba_0.6_Sr_0.4_TiO_3_ nanopowders were successfully prepared. A schematic diagram of the fabrication of Ba_0.6_Sr_0.4_TiO_3_ nanoparticles is shown in Figure S1.

### 2.3. Preparation of Ba_0.6_Sr_0.4_TiO_3_@DA Nanoparticles

First, the surface of BST was treated with hydroxyl groups. The Ba_0.6_Sr_0.4_TiO_3_ nanopowders were dispersed in a mixed solution (ethanol and water) with a ratio of ethanol to water of 1:1, stirred and ultrasonically dispersed for 4 h, and dried for 12 h. Then, the treated Ba_0.6_Sr_0.4_TiO_3_ nanopowders were added to a dopamine buffer solution (dopamine hydrochloride), and Mixture E was obtained by ultrasonic stirring for 10 h. Finally, Mixture E was centrifuged and dried, and the Ba_0.6_Sr_0.4_TiO_3_@DA nanoparticles were successfully obtained. Chemical surface modification of inorganic nanoparticles can improve the uniformity of nanocomposites [37]. The distribution of the diameters of Ba_0.6_Sr_0.4_TiO_3_ nanoparticles is shown in Figure S2. The diameter of the filler is mainly distributed in the 800 nm attachment. The TEM images of Ba_0.6_Sr_0.4_TiO_3_@DA NPs are shown in Figure S3.

### 2.4. Preparation of Boron Nitride Nanosheets

**Figure 1:**
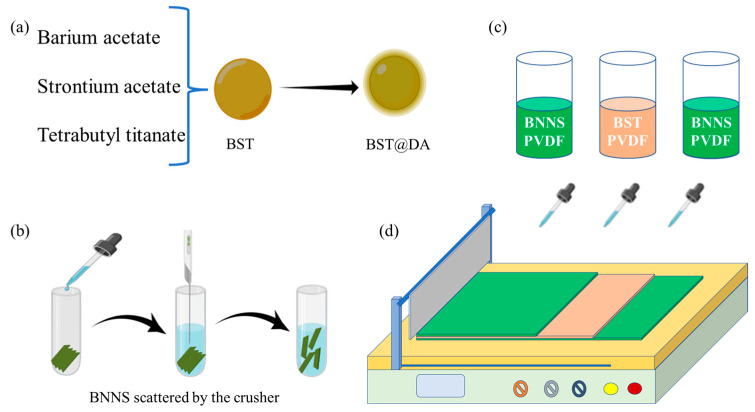
Schematic diagram of the preparation process of nanocomposite films. (**a**) Preparation of nano-filler, (**b**) Preparation of boron nitride nanosheets, (**c**) Preparation of mixed solution for tape casting, (**d**) The steps of casting method.

Boron nitride nanosheets (BNNS) were obtained by exfoliating hexagonal boron nitride powder (h-BN) by the chemical solvent ultrasonic method. The preparation process is shown in Figure 1. An amount of 1 g of hexagonal boron nitride powder was accurately weighed into a centrifuge tube, and absolute ethanol was added to the centrifuge tube. The centrifuge tube was placed under the ultrasonic probe, the height of the ultrasonic probe was adjusted, and the probe made as deep as possible toward the bottom of the centrifuge tube, without touching the wall of the centrifuge tube. The centrifuge tube mouth was sealed with parafilm to prevent solvent volatilization during ultrasonication. The power of the sonicator was set to 200 W. The mixture in the centrifuge tube was centrifuged after 2 h of uninterrupted operation of the needle tip sonicator. After centrifugation, the supernatant was discarded and the bottom pellet collected. The obtained precipitate was placed in a vacuum drying oven at 60 °C for 24 h to obtain boron nitride nanosheets (BNNS).

### 2.5. Preparation of Sandwich-Structured Composite Films

The sandwich-structured composite film material was prepared by way of layer-by-layer solution casting. The fabrication process of the composite films is shown in Figure 1. In this paper, 5 wt.% PVDF/BNNS-0 vol.% PVDF/Ba_0.6_Sr_0.4_TiO_3_@DA-5 wt.% PVDF/BNNS can be abbreviated as B0B, 5 wt.% PVDF/BNNS-1 vol.% PVDF/Ba_0.6_Sr_0.4_TiO_3_@DA-5 wt.% PVDF/BNNS can be abbreviated as B1B, 5 wt.% PVDF/BNNS-2 vol.% PVDF/Ba_0.6_Sr_0.4_TiO_3_@DA-5 wt.% PVDF/BNNS can be abbreviated as B2B, and 5 wt.% PVDF/BNNS-3 vol.% PVDF/Ba_0.6_Sr_0.4_TiO_3_@DA-5 wt.% PVDF/BNNS can be abbreviated as B3B. 

### 2.6. Characterization

In this study, the phase structures of inorganic nanoparticles and nanocomposites were analyzed using an X-ray diffractometer (XRD). The model of this X-ray diffractometer is Rigaku D/max-2200. The microstructure of the samples was analyzed by field emission scanning electron microscopy (FEI). The composition of the samples was analyzed using an energy dispersive spectrometer (EDS). The specific sample preparation method is as follows: For powder SEM sample preparation, the powder was dissolved in ethanol, and ultrasonic shock was performed for 30 min. The upper liquid was taken from the capillaries on the aluminum foil, dried and sprayed with gold, and observed. For sandwich-structured composite material SEM sample preparation, the composite material was placed in liquid nitrogen for quenching, the quenched sample was placed on the sample table, and the microstructure was observed after drying and spraying gold. Gold in a circle shape with a diameter of 1 cm was sputtered onto both sides of the composite films as the electrodes for the dielectric characterization. The dielectric constant and loss of the composite films were characterized using an Agilent E4980A. An Agilent precision impedance analyzer (E4980A, Agilent, Santa Clara, CA, USA) was also used for AC conductivity tests. The test frequency was 20 Hz-2 MHz, the temperature was room temperature, and the measurement circuit adopted a four-terminal double-lead mode. The AC conductivity of the sample was obtained by using the instrument to test the impedance of the sample. Gold in a circle shape with a diameter of 2 mm was sputtered onto both sides of the composite films as the electrodes for the characterization of ferroelectric parametron properties. The D-E hysteresis loops of the composite films were determined using a Radiant Premier II. A breakdown strength test was performed using a Precision Multiferroic ferroelectric tester from Radiant Technologies, USA. The test temperature range was room temperature, and the test frequency was 10 Hz. 

## 3. Results and Discussion

**Figure 2:**
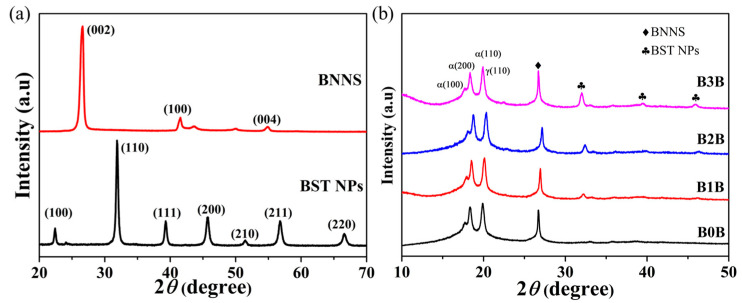
(**a**) XRD patterns of as-prepared Ba_0.6_Sr_0.4_TiO_3_ NPs and as-prepared BNNS. (**b**) XRD patterns of as-prepared different nanocomposites.

It can be seen from Figure 2a that BNNS mainly has a strong diffraction peak at 27°, which corresponds to the diffraction position of the (002) crystal plane. The peak is strong and sharp, indicating that the crystals of boron nitride nanosheets obtained after exfoliation are still highly ordered. The positions of the XRD peaks of the BST nanoparticles in Figure 2a are in perfect agreement with the standard Ba_0.6_Sr_0.4_TiO_3_ card, which proves that the perovskite-structured barium strontium titanate was successfully synthesized. No other peaks were generated in the XRD spectrum, indicating that the prepared BST nanoparticles were of high purity.

In order to analyze the phase structure of the sandwich-structured nanocomposites, the sandwich-structured nanocomposites with contents of 0 vol.%, 1 vol.%, 2 vol.%, and 3 vol.% of BST nanoparticles in the interlayer were characterized by X-ray diffraction, as shown in Figure 2b. The nano-nanocomposite material also appeared with a new diffraction peak, that is, the characteristic diffraction peak of BNNS at 27°, indicating that the crystalline structure of PVDF and BNNS was not destroyed in the composite of PVDF and BNNS. As can be seen from Figure 2b, for the prepared three-layer composite material, in addition to the diffraction peaks of PVDF, the characteristic diffraction peaks belonging to BST and the characteristic diffraction peaks of BNNS appear at the same time. It was demonstrated that sandwich-structured nanocomposite films were successfully prepared without significant damage to the crystallinity of BNNS and BST. The crystalline phase of PVDF in sandwich composites is mainly composed of the non-polar α phase and γ phase [38], in which the γ phase mainly originates from the quenching process of the composite film instantaneously transferred from a high temperature of 200 °C to ice water. The existence of nonferroelectric α and γ phases is beneficial to the dipole rotation of the composite film during the removal of the electric field, which helps to reduce the remnant polarization.

**Figure 3:**
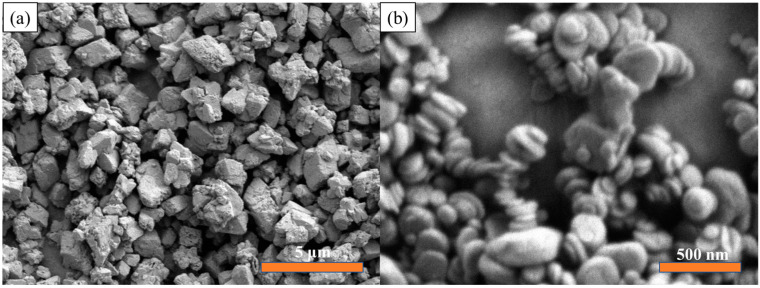
The SEM of (**a**) Ba_0.6_Sr_0.4_TiO_3_ NPs and (**b**) BNNS.

The SEM results of Ba_0.6_Sr_0.4_TiO_3_ NPs are shown in Figure 3a. The average diameter of Ba_0.6_Sr_0.4_TiO_3_ NPs is 500 nm, which means that the Ba_0.6_Sr_0.4_TiO_3_ NPs used in this paper can provide larger interfacial polarization and a higher dielectric constant. At the same time, they can also meet the high dielectric constant filler requirements for constructing high dielectric constant layers in “sandwich” composite dielectrics. The microscopic morphology of BNNS can be seen from the SEM image in Figure 3b. BNNS exhibits a lamellar structure, and the size in the plane direction is about 100 nm. The good dispersibility of BNNS is attributed to the strong polarity caused by the B-N chemical bond on the molecular plane, which not only helps its uniform dispersion in DMF but also its uniform distribution in the polar polymer matrix.

**Figure 4:**
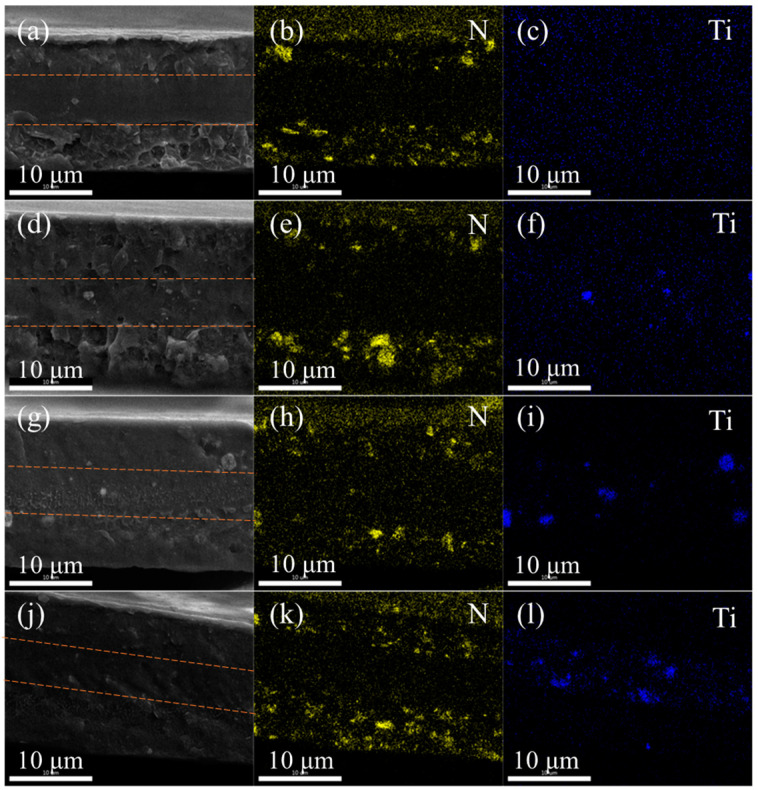
(**a**,**d**,**g**,**j**) Cross-sectional SEM image of the sandwich-structured nanocomposite with 0 vol.%, 1 vol.%, 2 vol.%, and 3 vol.% Ba_0.6_Sr_0.4_TiO_3_ NPs in the central layer, respectively. (**b**,**e**,**h**,**k**) EDS element (N) distribution of the sandwich-structured nanocomposite with 0 vol.%, 1 vol.%, 2 vol.%, and 3 vol.% Ba_0.6_Sr_0.4_TiO_3_ NPs in the central layer, respectively. (**c**,**f**,**i**,**l**) EDS element (Ti) of the sandwich-structured nanocomposite with 0 vol.%, 1 vol.%, 2 vol.%, and 3 vol.% Ba_0.6_Sr_0.4_TiO_3_ NPs in the central layer, respectively.

A schematic diagram of the sandwich-structured PVDF-based nanocomposite dielectric material is shown in Figure 4. Figure 4a,d,g,j are a cross-sectional scan of the sandwich-structured nanocomposite. The contents of BST NPs in the middle layer are 0 vol.%, 1 vol.%, 2 vol.%, and 3 vol.%, respectively. It can be seen from the cross-sectional scan that the nanofillers have good dispersibility in the polymer matrix of each layer. There are no defects such as separation or voids at the interface between different layers, indicating that the preparation process is good. There is no introduction of defects such as air, which is beneficial to the improvement in the dielectric properties of the three-layer structure material. The layered structure could not be clearly observed from the quenched cross-section of the sandwich composite film, so we performed an EDS surface scanning test on the sandwich composite. The distribution of the main elements (N, Ti) in the material is clearly observed, and the results are shown in Figure 4b–f,h,i,k,l, showing a uniform layered distribution. The Ti element is concentrated in the middle part of the sandwich composite film. The N element (BNNS) is dispersed on both sides of the sandwich composite film. With the increasing volume percentage of Ba_0.6_Sr_0.4_TiO_3_ NPs in the interlayer, the Ti element is more and more enriched, which is completely consistent with the original design of the experiment.

**Figure 5:**
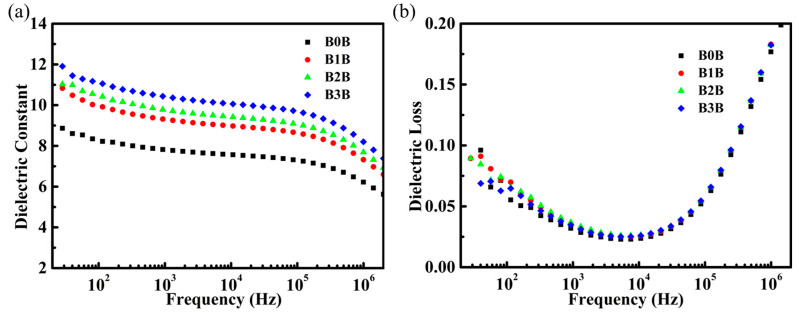
Frequency dependence of (**a**) dielectric constant and (**b**) dielectric loss of composites at room temperature.

The relationship between the dielectric constant and the dielectric loss as a function of frequency is characterized by the sandwich structure composite when the content of Ba_0.6_Sr_0.4_TiO_3_ NPs in the intermediate layer changes, as shown in Figure 5. As the content of Ba_0.6_Sr_0.4_TiO_3_ NPs in the interlayer gradually increased, the dielectric constant of the sandwich structure material also gradually increased. At 1000 Hz, the dielectric constant of Ba_0.6_Sr_0.4_TiO_3_ NPs with 3 vol.% volume fraction added is 10.71. The increase in the dielectric constant is mainly due to the high dielectric constant of Ba_0.6_Sr_0.4_TiO_3_ NPs. It can be seen from Figure 5b that although the content of Ba_0.6_Sr_0.4_TiO_3_ NPs in the interlayer is added to 3 vol.%, the dielectric loss of the sandwich composites is always maintained at a low level (tan δ < 0.04).

**Figure 6:**
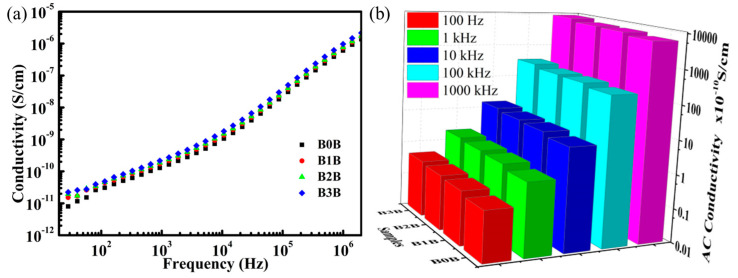
(**a**) AC conductivity of sandwich-structured nanocomposites with 0 vol.%, 1 vol.%, 2 vol.%, and 3 vol.% Ba_0.6_Sr_0.4_TiO_3_ NPs in the central layer. (**b**) Comparison of the AC conductivities of sandwich-structured composites with different Ba_0.6_Sr_0.4_TiO_3_ NP contents in the middle layer at 100 Hz, 1 kHz, 10 kHz, 100 kHz, and 1000 kHz.

Figure 6 shows the AC conductivity of sandwich-structured composite films. It can be seen from Figure 6a that the AC conductivities of the sandwich-structured composites are very low because BNNS can hinder the injection of charges at the electrodes and hinder the transport of charges in the polymer matrix. Low AC conductivity can reduce the leakage current of sandwich composites under a high field. On the other hand, the smaller conductivity loss of the sandwich composite will reduce the remanent polarization and increase the breakdown field strength of the composite. As shown in Figure 6b, the AC conductivity of the sandwich-structured composites exhibits a strong frequency dependence, which increases nonlinearly with increasing frequency. The conductivity of sandwich-structured composites increased with the increase in Ba_0.6_Sr_0.4_TiO_3_ NPs loading in the middle layer. However, the increase in conductivity of the sandwich-structured composites did not change by an order of magnitude. All sandwich-structured composites still have good insulating properties.

**Figure 7:**
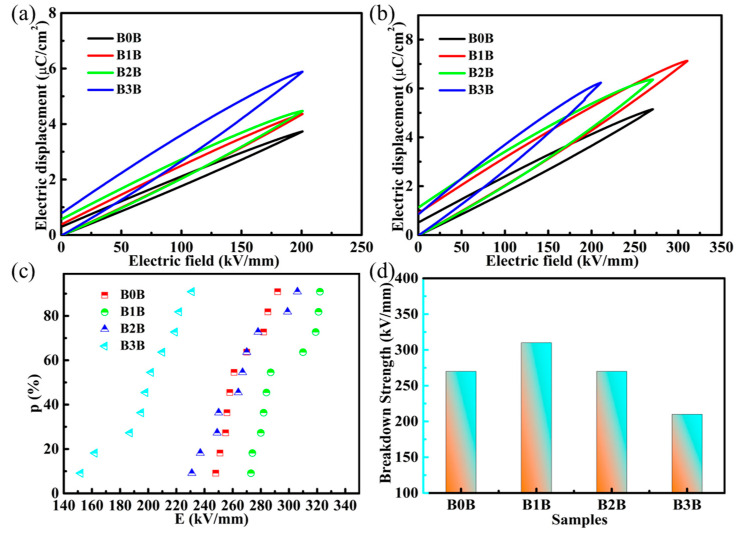
(**a**) Comparison of D-E loops of a series of sandwich-structured nanocomposites as a function of Ba_0.6_Sr_0.4_TiO_3_ NP content at an electric field of 200 kV/mm. (**b**) Comparison of D-E loops of a series of sandwich-structured nanocomposites as a function of Ba_0.6_Sr_0.4_TiO_3_ NP content at breakdown field strength. (**c**) Weibull plots of the sandwich-structured nanocomposites at varied Ba_0.6_Sr_0.4_TiO_3_ NP contents in the middle layer, (**d**) Weibull breakdown strength of the sandwich-structured nanocomposites as a function of Ba_0.6_Sr_0.4_TiO_3_ NP content in the middle layer.

The electrical displacement and electric field intensity curves were tested for pure PVDF polymer and sandwich nanocomposites. Figure 7a shows the D-E curve measured at an electric field strength of 200 kV/mm. It can be seen from Figure 7a that the maximum electrical displacement value of B0B at 200 kV/mm is only 3.72027 μC/cm^2^, which is due to the low dipole polarizability caused by PVDF with a low dielectric constant. With the increase in the content of Ba_0.6_Sr_0.4_TiO_3_ NPs in the interlayer, the maximum electrical displacement value gradually increased. At the addition of 3 vol.% Ba_0.6_Sr_0.4_TiO_3_ NPs, the maximum electrical displacement is 5.86947 μC/cm^2^, which is due to the gradual increase in the dielectric constant of the sandwich composites with the increase in Ba_0.6_Sr_0.4_TiO_3_ NPs. The remanent polarization of the sandwich structure also increases with the content of Ba_0.6_Sr_0.4_TiO_3_ NPs. At 0 vol.% Ba_0.6_Sr_0.4_TiO_3_ NP content, the remnant polarization is 0.31132 μC/cm^2^. With the gradual increase in the Ba_0.6_Sr_0.4_TiO_3_ NP content in the interlayer, the remanent polarization also increases gradually. At 1 vol.% Ba_0.6_Sr_0.4_TiO_3_ NP content, the remnant polarization is 0.39749 μC/cm^2^. When the addition of Ba_0.6_Sr_0.4_TiO_3_ NPs was 3 vol.%, the remnant polarization also reached the maximum value of 0.80512 μC/cm^2^, and the overall low remnant polarization of the sandwich composites is mainly attributed to the hindering effect of the outer BNNS. The existence of the outer layer of BNNS can effectively hinder the injection of electrons, thereby reducing the leakage current value of the material, which is also reflected in the AC conductivity of the composite material.

The D-E curve of the composite under the maximum electric field is shown in Figure 7b. It can be seen from Figure 7b that the sandwich-structured composite exhibits a nonlinear (ferroelectric) response to the external electric field at high fields, which is mainly due to the inherent ferroelectricity of PVDF and Ba_0.6_Sr_0.4_TiO_3_ NPs. The breakdown field strength of the prepared sandwich-structured nanocomposites also was analyzed, and the two-parameter Weibull distribution method was used for fitting [39].

(2) $$P\left(E\right)=1-\exp\left[-{\left(\frac{E}{E_{b}}\right)}^{\beta }\right]$$

In the above Formula (2), the value of *P*(*E*) is the cumulative failure probability of the electric field, and *E_b_* and *E*, respectively, refer to the breakdown field strength and the test breakdown field strength when the cumulative failure probability is 63.2% [40]. *β* is a shape parameter related to the discreteness of the experimental data. A high *β* value indicates that the nanocomposites are of high quality. The Weibull distribution diagram of the breakdown field strength is shown in Figure 7c, and Figure 7d is the variation of the Weibull breakdown field strength value with the content of Ba_0.6_Sr_0.4_TiO_3_ NPs in the intermediate layer. It can be seen from the figure that with the increase in Ba_0.6_Sr_0.4_TiO_3_ NPs in the sandwich structure material, the breakdown field strength of the three-layer composite material increases first and then decreases. When the Ba_0.6_Sr_0.4_TiO_3_ NP content was increased to 1 vol.%, the breakdown field strength of the three-layer composite was the largest, at 310 kV/mm. As the content of Ba_0.6_Sr_0.4_TiO_3_ NPs continued to increase, the breakdown field strength of the composites gradually decreased. With the highest addition of Ba_0.6_Sr_0.4_TiO_3_ NPs (3 vol.%), the breakdown field strength of the composite dropped to 210 kV/mm. With the increase in the content of Ba_0.6_Sr_0.4_TiO_3_ NPs in the middle layer, the sandwich structure composites showed a trend of first increasing and then decreasing: One reason is that after the content of Ba_0.6_Sr_0.4_TiO_3_ NPs exceeds 1 vol.%, the volume fraction of nanofillers may exceed the ideal percentage content that the polymer matrix can accommodate when the inorganic fillers are uniformly distributed. Too much filler leads to uneven distribution of Ba_0.6_Sr_0.4_TiO_3_ NPs in the PVDF matrix, or even agglomeration, which affects the improvement in the breakdown field strength. Another reason is the voltage divider between the different layers, which has been similarly reported in the work of others. In sandwich-structured nanocomposites composed of different dielectric layers, the electric field strength of the low-k layers is higher than that of the high-k layers. For the sandwich-structured nanocomposites in this study, the outer layer PVDF/BNNS can withstand higher electric field strengths than the middle layer PVDF/Ba_0.6_Sr_0.4_TiO_3_ NPs with a high dielectric constant due to its lower dielectric constant. When the overall three-layer composite is under the same voltage, with the increase in the Ba_0.6_Sr_0.4_TiO_3_ NP content in the intermediate layer, the dielectric constant of the intermediate layer increases continuously, so the voltage shared by the intermediate layer gradually decreases. When the content of Ba_0.6_Sr_0.4_TiO_3_ NPs is less than 1 vol.%, the intermediate layer will be broken down first due to the existence of Ba_0.6_Sr_0.4_TiO_3_ NPs, and the withstand voltage characteristics will be reduced. When the content of Ba_0.6_Sr_0.4_TiO_3_ NPs was greater than 1 vol.%, the voltage obtained by the outer layer continued to increase. Although the outer two layers have improved withstand voltage characteristics due to the presence of BNNS, the pressure drop has exceeded the withstand voltage range, causing the outer two layers to be broken down. The breakdown field strength of the final sandwich nanocomposite reaches the maximum value when the Ba_0.6_Sr_0.4_TiO_3_ NP content is 1 vol.%.

In addition to the above-mentioned breakdown field strength and polarization properties, D_m_-D_r_ also plays an important role in the performance of dielectric energy storage. Thus, the D_m_, D_r_, and D_m_-D_r_ of the complex were also calculated from the D-E curve, as shown in Figure 7a. Compared to B0B, B2B, and B3B, the D_m_ of the sandwich-structured composites is significantly improved due to the significantly higher breakdown field strength of B1B. At the same time, the D_r_ of B1B is kept at a very low level. Lower D_r_ is beneficial for high discharge energy density because it means that the stored energy can be effectively released during the discharge process. With the increase in Ba_0.6_Sr_0.4_TiO_3_ NP content, the D_m_-D_r_ of sandwich-structured composites first increased and then decreased. It should be noted that B1B exhibited the highest D_m_-D_r_ (6.2 C/cm^2^). The greatly improved breakdown field strength and D_m_-D_r_ in B1B composites may lead to simultaneous improvements in discharge energy density and energy efficiency. 

**Figure 8:**
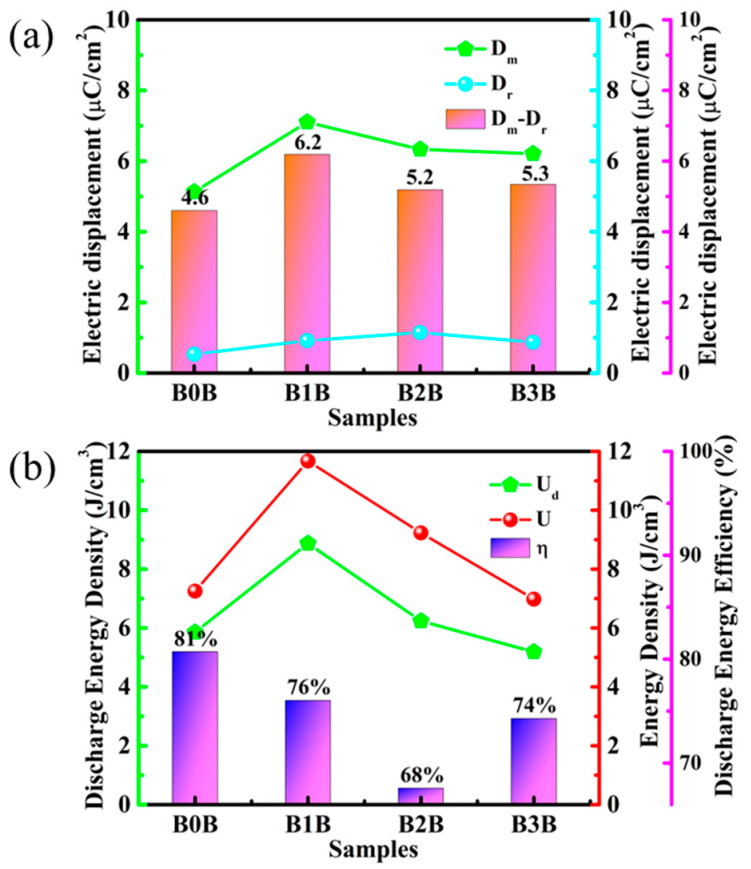
(**a**) Maximum electric displacement (D_m_), residual electrical displacement (D_r_), and the difference between residual electrical displacement and maximum electric displacement (D_m_-D_r_) as a function of Ba_0.6_Sr_0.4_TiO_3_ NP content in the middle layer of the sandwich-structured nanocomposites. (**b**) Maximum discharged energy density (U_d_), charged energy density (U), and charged-discharge energy efficiency (η) as a function of the different sandwich-structured nanocomposites with varied Ba_0.6_Sr_0.4_TiO_3_ NP contents in the middle layer.

In the above analysis, the Sawyer-Tower circuit was used to test the discharge energy density and charge-discharge efficiency of the sandwich structure composite at room temperature. The discharge energy density and energy efficiency of the sandwich-structured composites can be calculated from the D-E curves, and the results are shown in Figure 8b.

(3) $$\eta =\frac{U_{d}}{U}$$

In Formula (3), U_d_ is the released energy density of the material, and U is the stored energy density of the material [41]. For a more intuitive comparison, Figure 8b presents detailed data on the discharge energy density, charge energy density, and charge-discharge energy efficiency under the breakdown electric field for the four sandwich-structured composites. Although the maximum electrical displacement of B3B is larger than that of B2B, the discharge energy density of B2B is higher due to the higher breakdown field strength of B2B. At the same time, the higher D_r_ of B2B also leads to a significant drop in its energy efficiency. B1B exhibited the highest discharge energy density of 8.9 J/cm^3^ at a breakdown field of 310 kV/mm, an improvement over the energy storage performance data of PVDF obtained under the same conditions, which is shown in Figure S4. Although the energy efficiency of the composite gradually decreased with the electric field due to the ferroelectricity of PVDF and Ba_0.6_Sr_0.4_TiO_3_ NPs, the energy efficiency of B1B remained at 76% at an electric field of 310 kV/mm. Taking the discharge energy density and energy efficiency into consideration, B1B exhibits the best energy storage performance among a series of sandwich-structured composite dielectrics.

## 4. Conclusions

In this study, PVDF-based sandwich structure nanocomposites were prepared by a layer-by-layer solution casting method using PVDF as the matrix. In the sandwich-structured nanocomposites, 5 wt.% PVDF/BNNS was used as the outer layer of the three-layer structure, and 0 vol.%, 1 vol.%, 2 vol.%, and 3 vol.% PVDF/Ba_0.6_Sr_0.4_TiO_3_ NPs were used as the middle layer, respectively. The dispersion of BNNS by ultrasound and the coating of Ba_0.6_Sr_0.4_TiO_3_ NPs by dopamine promote the uniform distribution of nanofillers in the polymer matrix. SEM analysis showed that the layers of the sandwich-structured nanocomposites are tightly bonded to the other layers. EDS showed that the distribution of layers is very clear and the BNNS and Ba_0.6_Sr_0.4_TiO_3_ NPs are well dispersed. XRD analysis showed that the composites were mainly composed of non-polar α-phase and γ-phase, which was conducive to the rotation of the electric dipoles of the composites during the discharge process. The breakdown field strength of the sandwich structure composite with 1 vol.% PVDF/Ba_0.6_Sr_0.4_TiO_3_ in the middle layer reaches 310 Kv/mm. D-E curve analysis shows that the maximum discharge energy density of the sandwich-structured composite is 8.9, and the maximum discharge energy efficiency is 76%.

## Data Availability

The data presented in this study are available on request from the corresponding author.

## Supplementary Materials

The following supporting information can be downloaded at: https://www.mdpi.com/article/10.3390/polym15173642/s1, Figure S1. Schematic diagram of fabrication of Ba_0.6_Sr_0.4_TiO_3_ nanoparticles; Figure S2. The distribution of diameter of Ba_0.6_Sr_0.4_TiO_3_ nanoparticles; Figure S3. TEM images of Ba_0.6_Sr_0.4_TiO_3_@DA NPs; Figure S4. The energy storage performance data of PVDF obtained under the same conditions.
