# Supplementary material for: Tuning the Ferroelectric Response of Sandwich-Structured Nanocomposites with the Coordination of Ba0.6Sr0.4TiO3 Nanoparticles and Boron Nitride Nanosheets to Achieve Excellent Discharge Energy Density and Efficiency
Source: Polymers (Basel). 2023 Sep 4;15(17):3642. doi: 10.3390/polym15173642 (PMC10489975; doi:10.3390/polym15173642)
Supplement: Supplementary file 1 [file polymers-15-03642-s001.zip › polymers-2508239-supplementary.pdf]

Supporting Information

1 Ways of layer-by-layer solution casting

The high-temperature resistant glass plates were selected as the substrates prepared for sandwich-structured nanocomposites. The distance between the scraper of the casting machine and the glass plate was set. 5 mL of the mixture (BNNS/PVDF/DMF) onto a clean glass slide was dropped. The push rod of the coating machine pushes the scraper to move forward at a constant speed. The glass plates coated with the mixture (BNNS/PVDF/DMF) were then placed in an oven to dry to evaporate the DMF solvent. Then, in the same way, on the glass plates coated with the mixture ($Ba_{0.6}Sr_{0.4}TiO_3$@DA/PVDF/DMF), the mixture ($Ba_{0.6}Sr_{0.4}TiO_3$@DA/PVDF/DMF) with different volume fractions of barium strontium titanate were respectively coated as an intermediate layer and dried in an oven to evaporate the DMF solvent. Repeating the above procedure, the mixture (BNNS/PVDF/DMF) was applied as the third layer, which was placed in an oven to dry to evaporate the DMF solvent. After completing the appeal operation, the glass plates coated with the t sandwich-structured nanocomposites were dried in a vacuum drying oven at 100 °C for 12 hours to obtain different sandwich-structured nanocomposites.

The sandwich-structured nanocomposites were thermally treated as below. In order to remove the air in the composite material, the composite films were put in a vacuum drying oven keeping 200 °C for about 10 min with a vacuum environment of -90 kPa, for reducing defects.

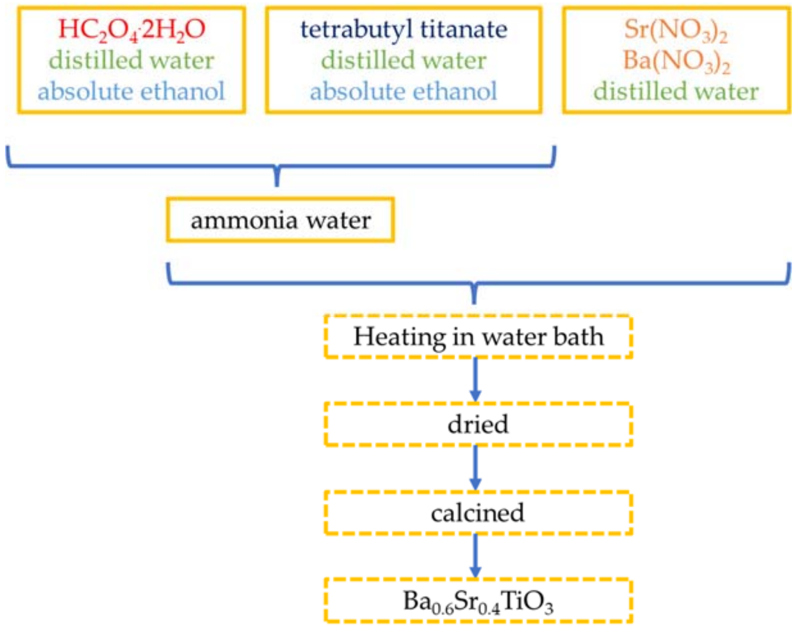

Figure S1. Schematic diagram of fabrication of $Ba_{0.6}Sr_{0.4}TiO_3$ nanoparticles

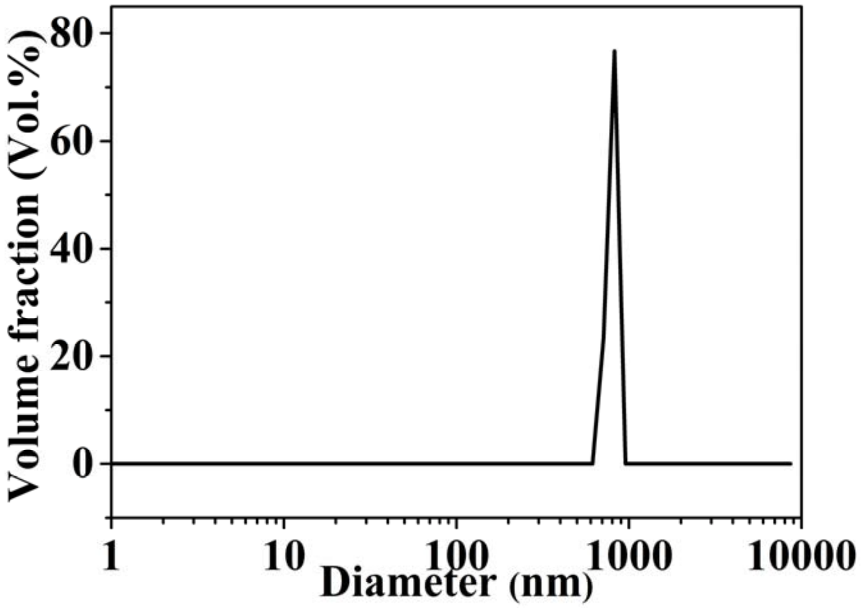

Figure S2. The distribution of diameter of $Ba_{0.6}Sr_{0.4}TiO_3$ nanoparticles.

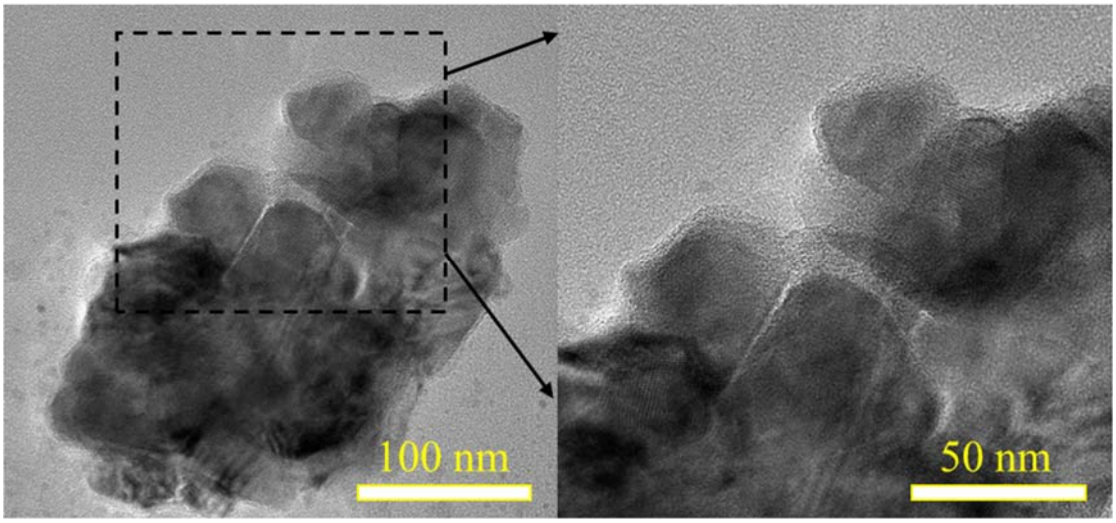

Figure S3. TEM images of $Ba_{0.6}Sr_{0.4}TiO_3$@DA NPs.

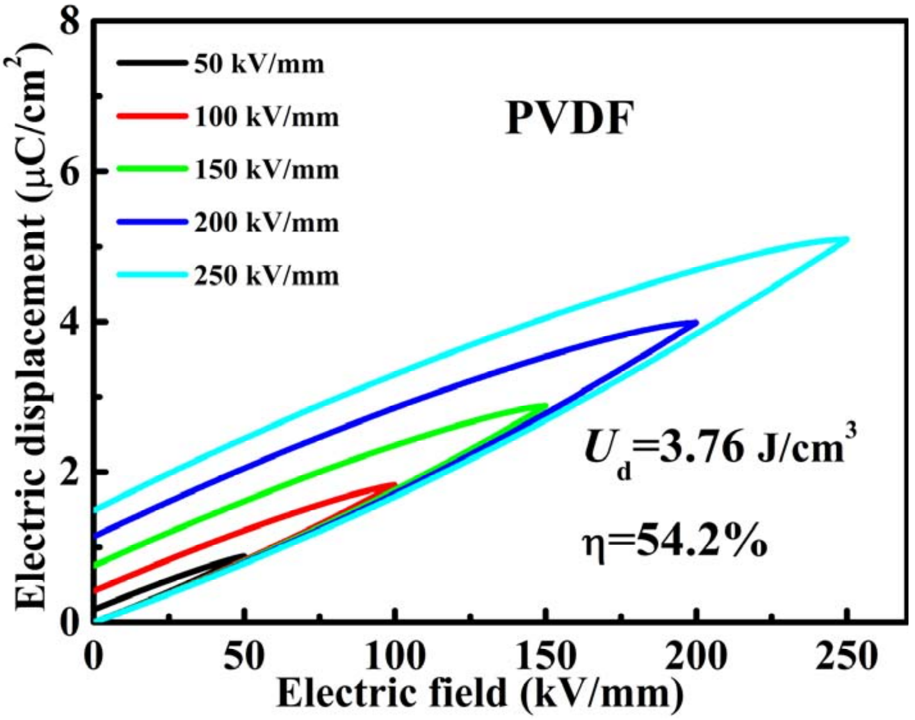

Figure S4. The energy storage performance data of PVDF obtained under the same conditions.
